# The ICGC ARGO data dictionary for standardizing global cancer clinical data

**DOI:** 10.1038/s41597-025-06068-4

**Published:** 2025-11-20

**Authors:** Hardeep K. Nahal-Bose, Peter Lichter, Ursula Weber, Lincoln D. Stein, Rosita Bajari, Linda Xiang, Edmund Su, Jon Eubank, Ciarán Schütte, Christina K. Yung, Mélanie Courtot

**Affiliations:** 1 Ontario Institute for Cancer Research (OICR), Toronto, ON M5G 1M1 Canada; 2 Division Molecular Genetics, German Cancer Research Center (DKFZ), Heidelberg, 69120 Germany; 3 Department of Molecular Genetics, University of Toronto, Toronto, ON M5S 3K3 Canada; 4 Element Biosciences, San Diego, CA 92121 USA; 5 Indoc Systems, Toronto, ON M5H 3W4 Canada; 6 Medical Biophysics Department, University of Toronto, Toronto, ON M5G 2C4 Canada; 7 Department of Computer Science, University of Toronto, Toronto, ON M5S 2E4 Canada

**Keywords:** Cancer models, Standards

## Abstract

The International Cancer Genome Consortium Accelerating Research in Genomic Oncology (ICGC ARGO) project is an international initiative to sequence germline and tumour genomes from 100,000 cancer patients across 13 countries and 22 tumour types. By integrating genomic data with comprehensive clinical information including treatment outcomes, lifestyle, environmental exposures and family history, ICGC ARGO aims to accelerate the application of genomic insights in cancer diagnosis, treatment and prevention. However, a major challenge is harmonizing clinical data from diverse tumour types worldwide. To address this, the ICGC ARGO Data Dictionary was developed to ensure consistent high-quality clinical data collection by defining a minimal set of clinical fields within an event-based data model to capture clinical relationships and support longitudinal data collection. Grounded in international standardized terminology, it is interoperable with other data standards such as Minimal Common Oncology Data Elements (mCODE). Its adoption by global initiatives such as the European-Canadian Cancer Network (EUCANCan) and the Marathon of Hope Cancer Centres Network (MOHCCN) underscores its broad impact on advancing precision oncology research.

## Introduction

### Challenges in the collection of cancer clinical data

Clinical data for cancer research is often scattered across multiple platforms and systems including clinical trials, electronic health records, pathology reports and laboratory results. Common types of clinical data that are core to cancer research such as cancer staging, biomarkers and drug dosages are often captured in unstructured formats and therefore require expensive and manual data abstraction and curation efforts, making it difficult to aggregate and analyze in a unified way. Adding to this challenge, these data frequently reside in “data silos” and are not connected, thereby hindering analysis of a patient’s cancer journey. An example of this is the American Society of Clinical Oncology’s (ASCO) data platform initiative CancerLinQ^1^ which is missing staging and molecular data information for 50% of its patient records, a problem attributed to a bottleneck in the curation of synoptic pathology reports and dictated texts^2^. Different systems and institutions may lack interoperability, such as using incompatible formats and terminologies for recording a patient’s data, making it difficult to exchange clinical data among institutions and hampering collaborative research efforts. Together, these challenges lead to incomplete or missing clinical data, which can skew analysis and limit the ability to fully understand a patient’s disease trajectory or response to treatment. Past analyses of large-scale cancer registries have shown heterogeneous differences in survival outcomes for patients with missing data, which in some cases has led to systematic over- and underestimations of survival for patients with missing data compared with those whose data is complete^3–5^.

### Challenges in ICGC ARGO

**Fig. 1:**
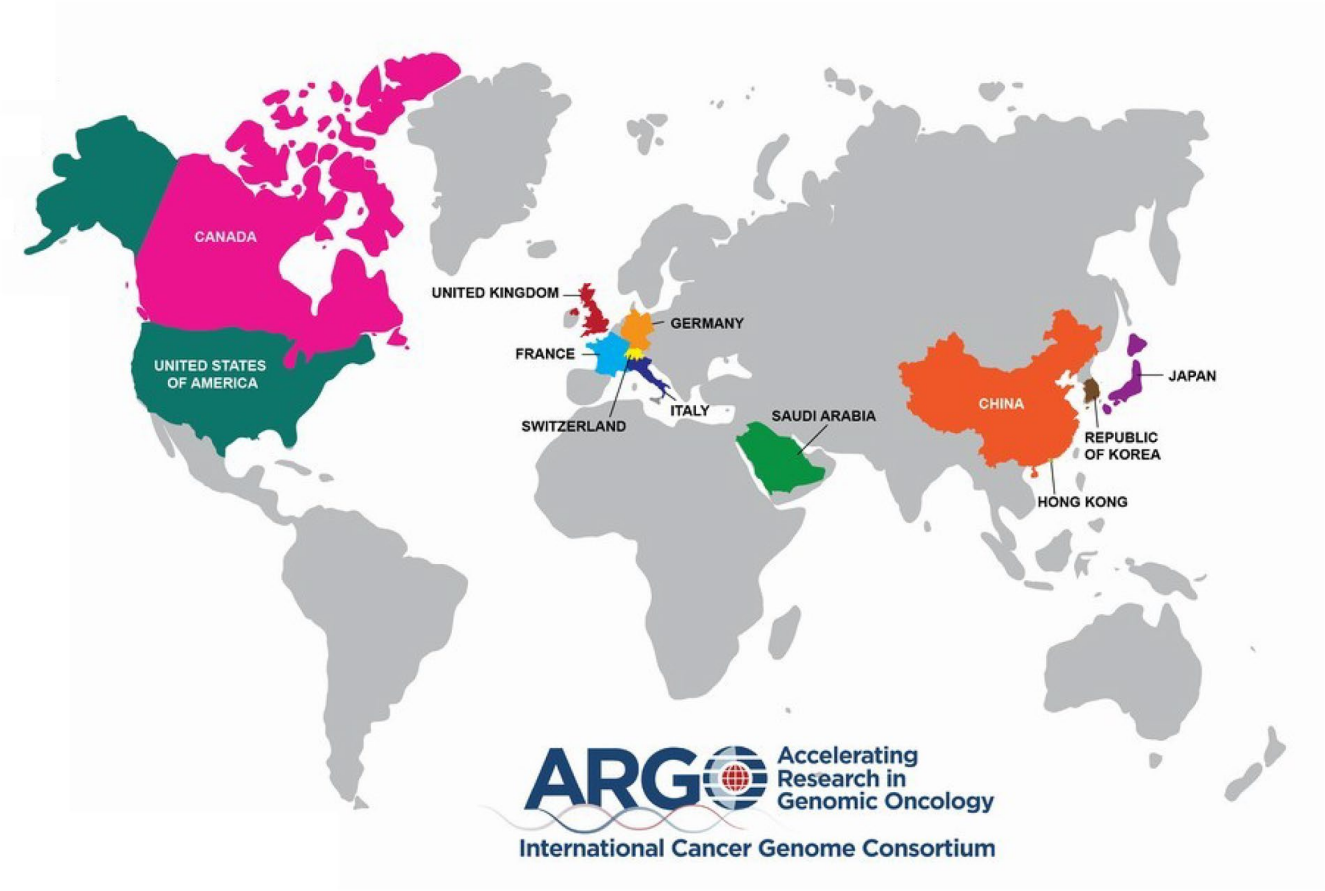
A world map showing the current global structure of ICGC ARGO member programs.

The ICGC ARGO project is a global initiative that operates in a complex environment that includes 26 member ARGO programs involving multiple cancers, as well as prospective and retrospective data that spans multiple countries and jurisdictions^6^ (Fig. 1). A significant challenge of the project is harmonizing large sets of genomic and clinical data from 100,000 patients across multiple cancer types and countries of origin to address key project goals and precision oncology research questions. The incidence of cancer, its response to treatment, and survival outcomes vary widely across different populations and regions, adding complexity to clinical data which must take into account environmental, lifestyle and genetic factors. In addition, a detailed and reliable portrayal of a cancer patient’s journey requires the integration of multiple dimensions of clinical and genomic information. As cancer unfolds longitudinally, its molecular characteristics and clinical manifestations change over time, which is critical for understanding the disease and designing effective treatments. For example, tracking somatic changes before and after treatment over many years can be complex to model in standard clinical data models (CDMs). Most standard CDMs are aimed at capturing short-term outcomes, whereas in cancer, a patient can live many years after treatment and requires capturing long-term outcomes such as survival rates, recurrence rates or the development of secondary cancers. Interoperability with other global initiatives facilitates the ability to make discoveries that can be accomplished only when multiple datasets can be merged and aggregated in a meaningful way. The ICGC ARGO Dictionary was built on lessons learned from the ICGC25K^7^ project, where for much of the genomic data obtained for 22,330 patients, key clinical information such as relapse type, length of disease-free interval, tumour staging and survival time were largely missing^8^. The complexity and diversity of challenges in ICGC ARGO require a specialized clinical data model that cannot be easily adopted from current CDMs. Therefore an early priority of the ICGC ARGO project was to establish an expanded, more comprehensive clinical data model to support the mission of the project. Since there is no current standardized oncology trial data model that spans all cancer types, data fields and values that explicitly meet the ICGC ARGO’s requirements were adopted^9^.

## Methods

### Data modeling process

The ICGC ARGO clinical data dictionary was jointly developed by the Ontario Institute for Cancer Research (OICR)^10^ and the ICGC ARGO Tissue & Clinical Annotation (TCA) Working Group^9^. The key requirement was to establish a minimal set of mandatory clinical parameters to be submitted for each donor, defined as the “core” set chosen to support key analytic tasks such as predictive biomarker discovery. These core parameters were specified on the basis of consensus within the working group, supplemented by engagement with clinicians, researchers and data custodians, and by subjectively weighing the ease of collection against the research value for each clinical field. The data model was designed to align with existing widely used standards, and was complemented by our own terminology for terms that did not have a common standard definition. We further defined an attribute for each term indicating whether the term was always “required”, or was “conditional”, meaning that a condition was needed for data capture of that term. For example, if a donor’s cancer was staged using the Ann Arbor staging system^11^, the clinical T, N and M fields would not be required since these fields are only relevant for the AJCC staging system, hence the fields related to the T, N and M values are marked as “conditional”.

**Fig. 2:**
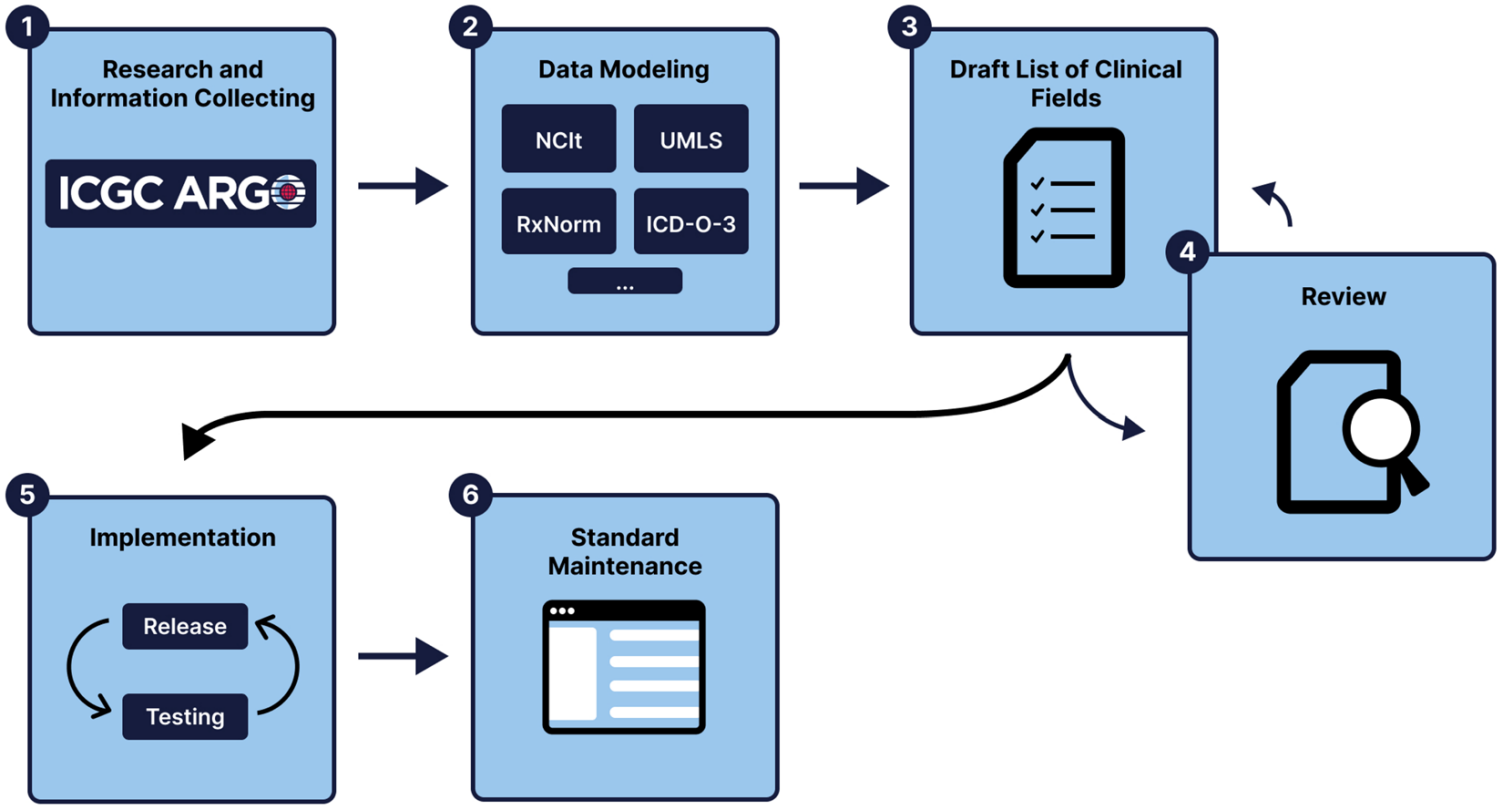
Overall methodology used to deliver the first version of the ICGC ARGO Dictionary.

The data modeling process involves six stages as shown in Fig. 2:

Stage 1 was an assessment stage and involved engaging with each ARGO program’s clinical coordinator and other data experts to gather information about cancer-specific fields collected within the scope of the program and each cancer type. This involved reviewing case report forms and use cases to aid in understanding when each clinical field was collected in a donor’s clinical timeline, such as for example specific biomarkers that were collected before and after treatment. Some clinical fields were collected across multiple cancer types, whereas other clinical fields were only applicable to specific cancer types.

Stage 2 involved modeling and developing concepts. Each clinical field was assessed to ensure that it was in line with the ICGC ARGO goals of defining a minimal set of clinical parameters applicable to all cancer types. Substantial differences in the collection of data for some clinical fields by different programs/cancer types, such as the use of different terminologies were noted, so adherence to clinical standards where possible was enforced. Examples include the NCI Thesaurus (NCIt)^12^, International Collaboration on Cancer Reporting (ICCR)^13^, Common Terminology Criteria for Adverse Events (CTCAE)^14^, Unified Medical Language System (UMLS)^15^, Logical Observation Identifiers Names and Codes (LOINC)^16^, Orphanet^17^ etc.

Stages 3 & 4: A draft list of proposed clinical fields was distributed to the TCA Working Group for review and feedback. Stages 3 and 4 were iterative processes, as they involved updating and reviewing clinical fields with relevant experts and addressing and resolving issues and concerns from members. This approach allowed different perspectives and prompted discussions to select the relevant core clinical fields that would best suit the goals and research questions of the ICGC ARGO project.

Stage 5 involved extending the core data model to incorporate new optional clinical fields specific to ICGC ARGO programs and enforcing validation rules where necessary. The clinical data model underwent rigorous testing in the ICGC ARGO Platform’s clinical submission system using various use cases. Several ARGO programs tested the model using their own clinical data, and proposed revisions were discussed and reviewed by the TCA Working Group to establish a robust and comprehensive clinical data model.

In Stage 6 the clinical data model was released for production purposes. It is periodically reviewed and updated as new treatments, medical concepts and diagnostic codes evolve, and issues that require changes, additions or deletions are identified. This maintenance involves regular collaboration among the Clinical and Metadata Working Group^18^, clinical experts, data custodians and database administrators. Feedback from user communities is monitored via a dedicated helpdesk maintained by the OICR Data Coordination Centre (DCC), and the dictionary is updated as needed.

### Dictionary viewer

**Fig. 3:**
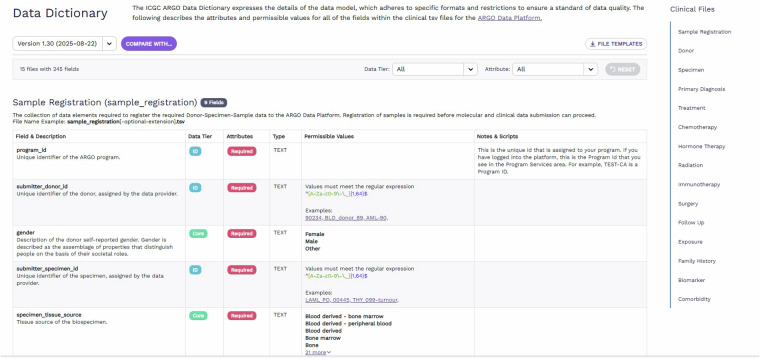
Interactive ICGC ARGO Dictionary Viewer.

As part of this effort, we developed a user-friendly interactive dictionary viewer located at https://docs.icgc-argo.org/dictionary where data producers and consumers are able to view the details of the data model including each schema and its details such as field descriptions, data tier and attributes, as well as permissible values and additional guidance for submission (Fig. 3). A key feature of the dictionary viewer is that it not only shows different versions of the dictionary but also allows comparisons between different versions by highlighting the changes between two different versions.

The dictionary viewer has been adopted by other groups for their use. One prominent example is EMBL-EBI’s CancerModels.org which hosts their Metadata Dictionary using ARGO’s Dictionary Viewer code at https://www.cancermodels.org/validation/dictionary. Given the popularity of the dictionary viewer, we are currently working on making the dictionary viewer a standalone component that can be easily imported and deployed by users in the near future. This will be done within OICR’s Overture suite, a collection of open-source software tools designed to be reusable and scalable to help create platforms for researchers to manage, share and access genomic data^19^.

## Results

### ICGC ARGO data dictionary

The ICGC ARGO clinical data model was finalized in June 2020 with the first version of the data dictionary released to the community. Since its inception in 2020, there have been 25 updates to the model. Each field in the dictionary has a data tier and an attribute classification that helps identify which fields are necessary for clinical data completion. There are three types of data tiers:

1. ID: Indicates that a field is a unique identifier, or a primary or foreign key that is used for cross file validation.
2. Core: Indicates that a field is part of the mandatory set of clinical fields that must be submitted.
3. Extended: Indicates that a field is optional but not required for clinical data completion.

There are two types of attribute classifications:

1. Required: Indicates that a field must be provided in the submission file for clinical data completion.
2. Conditional: The field must meet certain conditions, depending on the value of another field. When a “core” field is paired with a “conditional” attribute, this type of field is only required if the conditional requirements are met.

The ARGO data model is event-based and donor-centric and as of version 1.28 it consists of fifteen entities in total with 79 core fields and 113 extended (optional) fields across the areas of sample registration, donor, specimen, primary diagnosis, treatment, chemotherapy, surgery, hormone therapy, radiation, immunotherapy, follow up, biomarker, exposure, family history and comorbidity. Of the 79 core fields, 25 are required, whereas 54 are conditional, meaning that the field is only required if the conditional requirements are met.

There are six schemas for Sample Registration, Donor, Specimen, Primary Diagnosis, Treatment and Follow Up. Complete clinical data means that a donor has a valid value submitted for all fields labelled “core” in the data dictionary, with a donor having a minimum set of data submitted in these clinical files:

- **Sample Registration**: Defines the minimal set of clinical information required to register a sample. Importantly, the relationships between entities are maintained across all data submissions, as they are fundamental to data integrity across the ARGO Data Platform^20^. Thus, during sample registration, each Donor, Specimen, and Sample entity is assigned an ICGC ARGO ID that maps to a program’s internal identifier.
- **Donor**: A collection of clinical fields related to a specific donor, such as vital status, survival time, genetic disorders and primary site of cancer(s). Birth sex is not collected since it is determined from molecular data analysis.
- **Specimen**: Defines a set of clinical fields specific to a donor’s tumour or normal specimen that was used for genomic analysis, such as pathological staging, tumour grading and histology.
- **Primary Diagnosis**: Contains clinical fields related to a donor’s primary diagnosis such as cancer type, age at diagnosis, clinical staging and presenting symptoms.
- **Treatment**: Basic information about the type of treatment(s), start and duration of treatment, response to treatment and toxicity and adverse event clinical information.
- **Follow up**: Details about a donor’s follow up including disease status, type of relapse, and recurrence or posttherapy staging are included.

In addition, there are five schemas for submitting detailed treatment information for Immunotherapy, Chemotherapy, Hormone Therapy, Surgery and Radiation. These tables are required depending on the value submitted in the “treatment_type” field in the Treatment table:

- **Chemotherapy**: Details about each chemotherapy drug administered such as drug name, cumulative dose, and whether there was a dose reduction.
- **Hormone Therapy**: Details about each hormone therapy drug administered such as drug name and cumulative drug dose.
- **Immunotherapy**: Details about the type of immunotherapy delivered, drug name and cumulative drug dose information.
- **Radiation**: Details about radiation treatment such as the type of radiation, anatomic site of radiation administration and dosage.
- **Surgery**: Details about surgery type, whether a specimen was resected during surgery, information about whether margins were involved, not involved or not assessed, tumour dimensions and invasion.

There are also four optional schemas for collecting clinical variables that impact cancer risk and progression:

- **Exposure**: This includes clinical fields related to alcohol history, tobacco use, exercise frequency and certain diet consumption fields.
- **Family history**: This includes clinical fields related to a donor’s family history of cancer, such as relatives and the type of cancer they were diagnosed with and their vital status and survival time.
- **Comorbidity**: Clinical details about a donor’s comorbidities, including prior malignancies and the age at which they were diagnosed.
- **Biomarker**: A collection of clinical fields related to biomarkers that can be linked to different clinical events or time points.

**Fig. 4:**
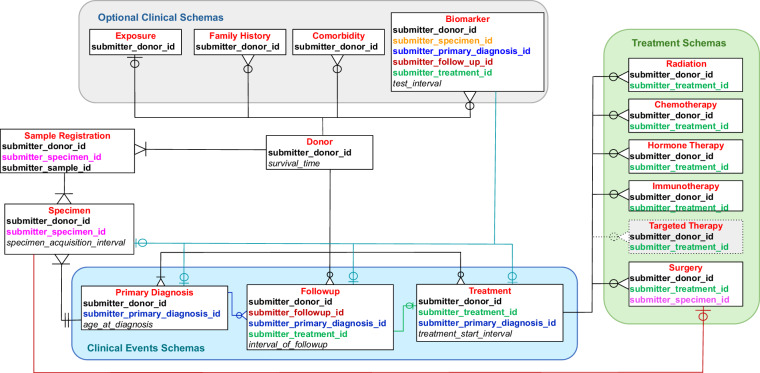
Entity-Relationship Diagram showing relationships between different schemas in the ICGC ARGO Dictionary. A new schema for “Targeted Therapy” will be added as part of future development.

The ARGO data model is encoded in JSON^21^ with validation tests built into the schemas. Each core or extended field that has a value type of “text” has a list of allowable terms called a code list. These code lists are based on international standardized terminology whenever possible and are stored in JSON format in the data model. If the code list is repeated in several fields or is quite long, the code list is stored in a central JSON file that is referenced in the schema. Otherwise, if the code list is short and unique to a field, it is included directly in the schema in the “codeList” key. The current version of the ARGO dictionary can be found in the ICGC ARGO GitHub repository. In addition, the data model establishes semantic relationships between different entities and fields by specifying a foreign key in each JSON schema. This ensures linkages between donors, specimens and related clinical events are accurately and consistently represented (Fig. 4). Examples include:

- In ICGC ARGO, a sample is considered the molecular extract and a specimen is the tissue from which the sample is derived. Each sample (submitter_sample_id) submitted in the Sample Registration schema must belong to a specimen (submitter_specimen_id). Since the time at which a specimen was taken from the patient is recorded (specimen_acquistion_interval), it is possible to submit multiple samples taken at different times.
- Each specimen (submitter_specimen_id) must belong to a single donor (submitter_donor_id).
- A specimen (submitter_specimen_id) must be linked to a donor’s specific primary diagnosis (submitter_primary_diagnosis_id).
- A follow up event (submitter_follow_up_id) can be linked to a specific treatment (submitter_treatment_id).
- A data submitter can denote multiple treatment types as a treatment regimen, such as combination chemotherapy and radiation therapy.
- If a specimen (submitter_specimen_id) was resected during surgery, it can be denoted in the surgery submission file.
- A biomarker test can be linked to any clinical event which can include primary diagnosis, treatment or follow up. For example, if a biomarker test is associated with a biopsied specimen taken during surgery, it can be linked to that specific submitter_specimen_id.

### Key features

#### Standardized terminology

The use of standardized terminology is important, especially since it is within the scope of the ICGC ARGO project which involves international programs and different languages. Where possible, each clinical field is annotated with references to relevant external standards, such as terms from controlled vocabularies. For some fields, we created our own terminologies for terms that did not have a common standard definition. Examples of standardized vocabularies used include the following:

- The ICGC ARGO Dictionary validates submitted drug names and drug identifiers against publicly available drug databases including RxNorm^22^, PubChem^23^, KEGG^24^ and the NCI Thesaurus. As a global initiative, drugs often have different names in different countries and languages, and cross-validation of the submitted drug name enables this. This also allows data submitters to submit investigational drugs.
- The American Joint Committee on Cancer Staging Classifications (AJCC)^25^ is used to derive staging classifications and stage groups for different editions of the AJCC.
- The World Health Organization International Classification of Diseases, 10th Revision (ICD-10)^26^ and whenever possible, the International Classification of Diseases for Oncology, 3rd edition (ICD-O-3)^27^ are used to specify the cancer type, primary site, topography and histology.

**Fig. 5:**
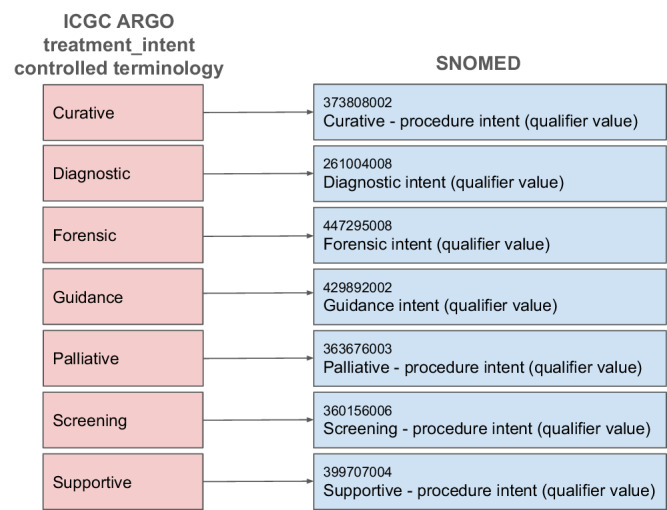
Example of field to ontology mapping between ICGC ARGO “treatment_intent” controlled terminology and SNOMED concepts.

The fact that the ICGC ARGO dictionary is based on standardized terminology may not be apparent when the dictionary is reviewed in the dictionary viewer at https://docs.icgc-argo.org/dictionary because it does not include references for the individual terms in each code list. This led Frid *et al*.^28^ to make the incorrect assumption that the ICGC ARGO clinical dictionary only uses standardized vocabulary for one variable^28^. However, as explained above, most of the terms in the ICGC ARGO dictionary use standardized terminology to ensure interoperability. (Table S1). Detailed references to standardized terminologies, which will be incorporated into the dictionary viewer as part of future development/improvements are provided in a downloadable table on Figshare^29^. Fig. 5 shows an example of how the controlled terminology for the “treatment_intent” field maps to SNOMED Overall, 60% of the core clinical fields are derived from standard terminology, while 57% all dictionary fields (core and extended) are derived from standard terminology while the remaining fields are complemented by our own terminology for terms that did not have a common standard definition. In addition to using standardized terminology, 60% of the fields’ descriptions align with other standards with well-defined semantics such as NCIt, LOINC. For example, the source of the field description for “percent_inflammatory_tissue” (“A quantitative measurement of the percent of a sample that is positive for inflammatory markers, including the presence of capillary dilatation, edema and increased leukocytes”) is from NCI Thesaurus Code C159479. This reduces ambiguity and helps optimize the data model for natural language understanding.

#### Data validation and verification

In accordance with the Good Clinical Data Management Practices (GCDMP)^30^ guidelines, the ICGC ARGO clinical dictionary and clinical submission system employ both cross-field and cross-file validation checks to ensure data integrity and to improve data quality by identifying inconsistencies such as data that are out of range or discrepant^31^. Cross-field validation checks are encoded in JavaScript in the data model and are executed at the time of clinical data submission to the ICGC ARGO Data Platform. An example of a cross-field validation check involves validating the text submitted value in the “drug_name” field against RxNorm. If an unrecognized drug name is submitted, an error message is reported. Another example of a cross-field validation involves validating the submitted “stage_group” against the submitted staging system in the “pathological_staging_system” field to ensure that the submitted stage group belongs to the staging system specified. If the stage group does not belong to the staging system submitted, an error message is reported indicating this. Cross-file validations are in place to ensure that clinical events align and exist correctly. Each clinical event is identified by a unique identifier field and a time interval field; these are validated across the submission files to ensure accuracy and correctness. For example, a treatment clinical event cannot be submitted after a donor has been indicated as deceased in the “vital_status” field from the Donor schema. Another example of cross file validation is preventing a field related to a tumour specimen (eg. “tumour_grade”) to be submitted for a normal specimen. When a submitted value is identified as incorrect, the value is flagged with a clear, concise error message notifying the data submitter of the error.

#### Longitudinal data collection

**Fig. 6:**
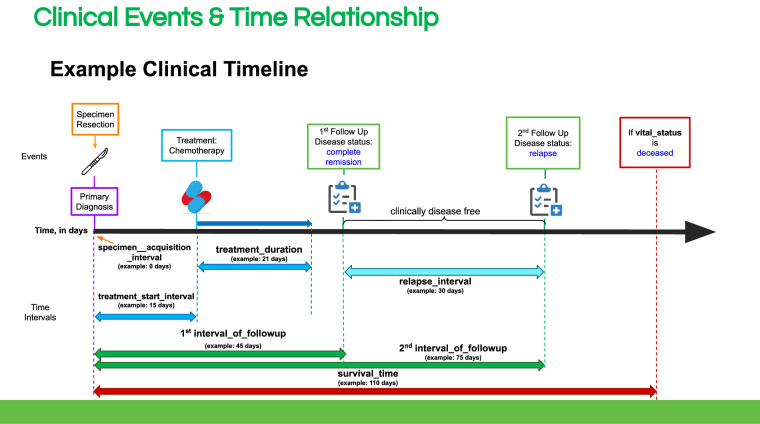
Example clinical timeline demonstrating how time intervals are used to validate longitudinal clinical data submissions in ICGC ARGO.

A key feature of the ICGC ARGO clinical data model is that it enables the collection of longitudinal data. The age at primary diagnosis (“age_at_diagnosis” field) is used as the reference time point and is used to calculate all time interval clinical fields, whereas the day the donor dies (“survival_time” field) is the clinical endpoint (Fig. 6). Time interval fields allow the data submitter to submit multiple events at different time points, and are used in place of actual dates to allow the data to stay in the open-access tier by enhancing data anonymization.

The identifier fields allow the data submitter to link different clinical events together. For example, a follow up event can be linked to a specific treatment, a surgery can be linked to a resected specimen, or a biomarker can be linked to when it was assessed, such as at the time of primary diagnosis. (Fig. 4) This allows the genomic profile of specimens collected at different time intervals - for example, before and after treatment, to be tied to clinical data such as staging, disease status and biomarkers.

The longitudinal data collection feature of the ICGC ARGO clinical data model is a key differentiator from traditional cancer data models. Most existing cancer data models rely on only baseline or endpoint data, such as genomics from just one sample, or response to just the initial treatment. The temporal modeling design of the ICGC ARGO data model enables it to capture the full spectrum of the disease providing a more granular perspective that includes both the timing and sequence of genetic mutations, treatments and outcomes. This allows for a much deeper understanding of how cancer evolves and has enormous potential to provide new insights into understanding treatment resistance, long term outcomes and facilitating development of predictive biomarkers, thus paving the way for more effective treatments based on a patient’s disease trajectory rather than a one-size-fits-all approach to treating cancer.

## Discussion

The ICGC ARGO dictionary provides numerous benefits, including improved data consistency, enhanced data sharing capabilities, and the ability to perform cross-study analyses. By standardizing data annotation, the dictionary reduces ambiguities in interpreting cancer genomics datasets, thus accelerating research and enabling more reliable conclusions. To prevent unnecessary duplication of efforts and promote interoperability, we reviewed several existing standards and models to assess whether we could adopt or leverage the strengths of existing terminologies. This included resources such as the Observational Medical Outcomes Partnership Common Data Model (OMOP)^31^, the Patient Centred Outcomes Research Network Data Model (PCORNet)^32^ and the Clinical Data Interchange Standards Consortium’s Study Data Tabulation Model (CDISC SDTM)^33^. All of these CDMs use terminologies that are widely used in various clinical systems but do not adequately cover cancer diagnoses, treatments and follow up events, and therefore require more granularity for ICGC ARGO’s purposes. The OMOP model can accommodate longitudinal data, but not specific clinical events that are captured in ICGC ARGO use cases. (OMOP is actively developing an Oncology Extension which aims to improve support for future cancer studies^34^.) The CDISC SDTM is mainly tailored for clinical trial data and is not structured to support post-trial follow-up or long-term outcomes. PCORNet does not use some of the standard terminologies used in cancer such as RECIST^35^ or AJCC staging. PCORNet has also not yet been implemented outside the United States, leaving its potential for international application uncertain^36^. Although these CDMs were not adopted for the purpose of ICGC ARGO, we leveraged some of their strengths and constructed a CDM that was comprehensive for cancer research and interoperable with other CDMs such as the Minimal Common Oncology Data Elements (mCODE)^37^. As an example, we took into account the ability to accommodate longitudinal clinical data from OMOP, the structured approach to collecting clinical data from CDISC which enhances interoperability, and the enforcement of mapping data to controlled vocabularies and centering of the data model on the patient entity from PCORNet.

We developed a comprehensive data model aimed at collecting highly annotated clinical data that can be integrated with uniformly analyzed genomic data. A minimal set of clinical fields that are feasible to collect, balanced by data validation checks and the use of controlled terminology to minimize unstructured free-text enables high quality clinical data collection and interoperability.

### Interoperability

The ICGC ARGO clinical data model is interoperable with several other clinical data models such as mCODE, GA4GHPhenopackets^38^ and 1+Million Genomes Initiative Minimal Dataset for Cancer (1+MG-MDC)^39^ and is used by several funded projects including the European-Canadian Cancer Network (EUCANCan)^40^, the EuCanImage project^41^ and the Marathon of Hope Cancer Centres Network (MOHCCN)^42^. Its intuitive structure and familiarity among clinical partners enables efficient data encoding and promotes harmonization across datasets and institutions.

**Fig. 7:**
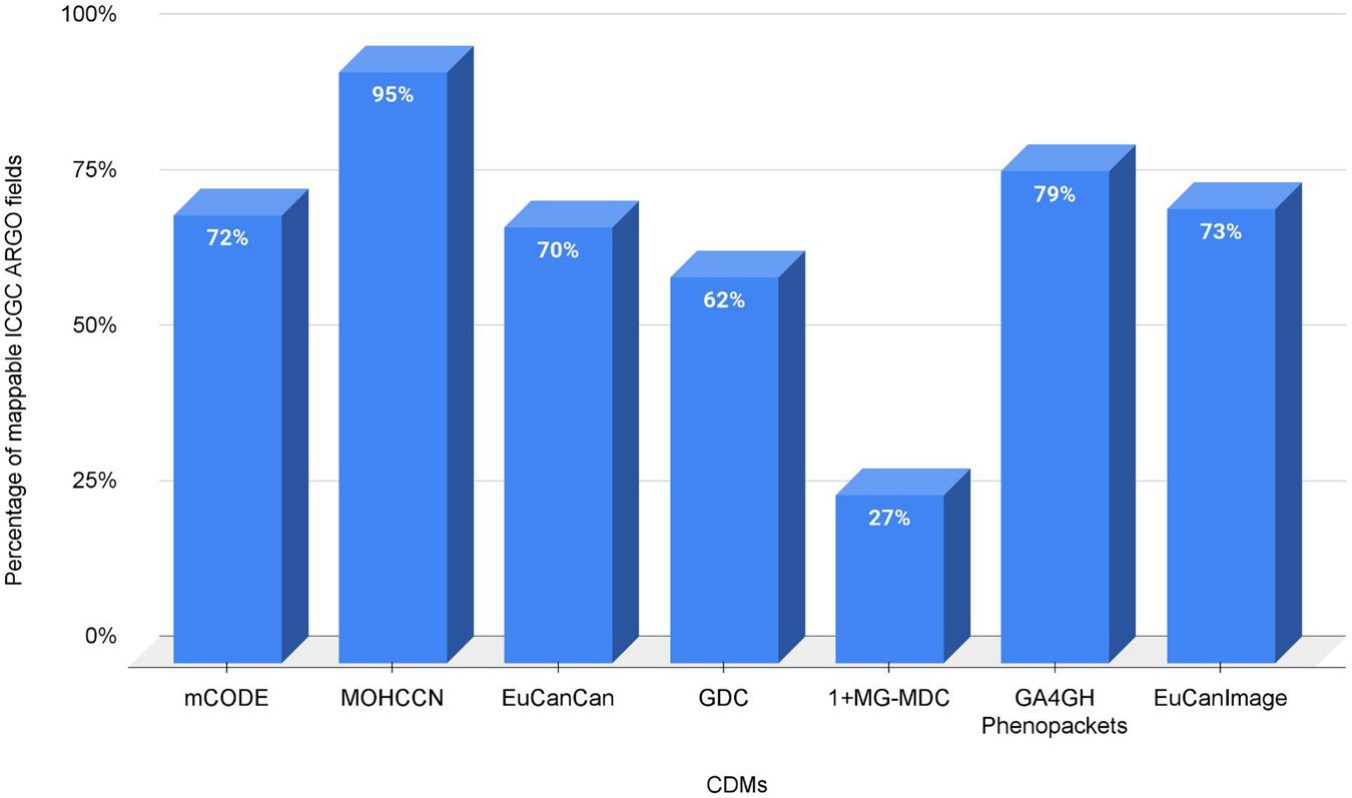
Percentage of ICGC ARGO fields that map to other oncology-focused CDMs.

A key strength of the ICGC ARGO data model is its high proportion of interoperable fields with other oncology-focused CDMs, reflecting strong alignment in both data structure and semantics. (Fig. 7) (see Table 2 deposited in Figshare^43^). This interoperability minimizes the need for complex data transformations, preserves meaning, and supports the reuse of scalable applications across multiple datasets. However, it is important to recognize that interoperability assessments can often be influenced by differences in data scope and domain complexity^44^. For example, while some models such as mCODE and 1 + MG-MDC incorporate both clinical and genomic data, the ARGO data model is focused exclusively on clinical data and its relationship to biological specimens, with genomic data managed separately. As a result, CDMs such as mCODE may appear to have lower interoperability with the ICGC ARGO data model, but this apparent reduction reflects differences in domain coverage rather than deficiencies in the CDM’s actual interoperability capabilities. Here we discuss the data model’s interoperability with seven other commonly-used models for oncology data.

- **Minimal Common Oncology Data Elements (mCODE)**: mCODE is an initiative developed by MITRE and the American Society of Clinical Oncology (ASCO) that aims to identify the cancer data elements required for analyzing treatment across different electronic health record systems^37^. OICR consulted with the mCODE team during the early phases of the ICGC ARGO dictionary development process to explore the interoperability of the two clinical data models. Mapping of version 1.28 of the ICGC ARGO clinical data model to mCODE version STU 4.0^45^ shows that 72% of the ICGC ARGO core clinical fields have a one-to-one match with an mCODE field, or can be partially derived from mCODE’s controlled vocabulary. (see Table 3 deposited in Figshare^46^). Conversely, mapping in the opposite direction showed that only 52% of mCODE’s 170 required fields map to fields in ICGC ARGO. However, this asymmetry is attributable primarily to mCODE’s inclusion of genomic data fields which account for 49% of the unmatched fields. When genomic data fields are excluded and only clinical data fields are considered, the interoperability rate of mCODE required fields mapping to ARGO increases to 70%, demonstrating that the two CDMs are highly interoperable with each other. Among the remaining 30% unmatched fields, most are related to clinical data not collected in ICGC ARGO such as cancer risk assessments, body surface area, patient details such as name, address, language and contact details, Karnofsky and Lansky performance status scales (the ECOG performance scale is used in ICGC ARGO), and imaging scoring details.
- **The Marathon of Hope Cancer Centres Network (MOHCCN)**: The Marathon of Hope Cancer Centres Network (MOHCCN) is a Canadian initiative aimed at creating Canada’s largest cancer case database containing 15,000 cancer genomes with related clinical data within five years, to enable new discoveries for the benefit of cancer patients^47^. The MOHCCN’s Data Policy & Standards Committee recommended that the ICGC ARGO Data Dictionary be used to describe clinical and other health-related fields since alignment of the ICGC ARGO clinical fields with mCODE and its FHIR implementation indicated good overlap between the two data models and promoted the integration and interoperability of projects using mCODE^47^. Mapping between version 1.28 of the ICGC ARGO data model and version 3.1 of the MOHCCN data model^48^ shows that 95% of ICGC ARGO core clinical fields can be mapped to a field in the MOHCCN data model. Using the current MOHCCN clinical data model (version 3.1) the MOHCCN has collected complete clinical data for 3,549 cases for their Gold Cohort as of May 14, 2025^49^.
- **EuCanImage**: The goal of the EuCanImage project is to build a federated European cancer imaging platform that will advance the use of artificial intelligence (AI) in oncology^41^. The ICGC ARGO clinical dictionary was selected as the basis of the EuCanImage data model of clinical variables^50,51^. A working group composed of European Genome-phenome Archive (EGA) partners, clinical experts, and the ICGC ARGO data model curator evaluated a list of clinical data variables for eight distinct use cases from liver, colorectal, and breast cancer types involved in the EuCanImage project. Their goal was to determine which parameters could be mapped to the ICGC ARGO clinical dictionary, identify any gaps based on input from clinical stakeholders, and explore the potential to expand the ICGC ARGO clinical dictionary to incorporate any missing clinical fields. An analysis of the use cases revealed that the ICGC ARGO clinical dictionary implemented 64% of the clinical variables used by the eight use cases, with an additional 9% added to the ICGC ARGO clinical dictionary in 2022^50^. A use case involving breast cancer highlighted by Frid *et al*.^28^ had the lowest overlap with the ARGO dictionary (58.1%), which was largely due to fields related to breast cancer molecular subtypes and the incorrect assumption that the model cannot differentiate diagnostic biopsy biomarkers from surgical biopsy biomarkers^28^. Current versions of the ICGC ARGO dictionary are tailored to fields that ARGO programs plan to submit to ICGC ARGO. The EuCanImage-specific breast cancer fields missing from the ICGC ARGO dictionary were not added because ARGO programs involved in breast cancer did not plan to collect and submit these fields. Additionally the use of foreign keys in each schema permits the ability to differentiate diagnostic biopsy biomarkers from surgical biopsy biomarkers.
- **GA4GH Phenopackets**: GA4GH developed the Phenopackets standard to provide a human and machine-readable way to structure phenotypic and clinical information about a patient or individual^38^. The GA4GH Phenopackets Cancer Task Team aligned variables in Phenopackets enabling the export of ICGC ARGO clinical data^52^. Mapping of version 1.28 of the ICGC ARGO data model against version 2.0 of the Phenopackets schema reveals that 79% of ICGC ARGO fields can be mapped to an element in the Phenopackets schema. While most of the core variables required by ICGC ARGO can be exported from Phenopackets, there are several limitations such as lack of clinical fields related to a follow up, or the ability to link clinical events to each other in the Phenopackets schema.
- **1+Million Genomes Minimal Dataset for Cancer (1+MG-MDC):** The 1+Million Genomes Initiative developed a minimal dataset encompassing 140 fields for the collection of cancer related clinical data and genomics metadata. Among these fields, 77 fields (55%) mapped to fields in ICGC ARGO^39,53^. Only 10% of the unmappable fields (6 fields) were related to cancer. Among the remaining unmappable fields, 60% were related to metadata associated with genomic files, alignment and variant calling software, and matching sample information - most of which is captured during genomic data submission and/or genomic data processing in ARGO rather than in the ARGO clinical data model. The other 30% of the unmappable fields were related to dates, which are not recorded in the ICGC ARGO; instead the interval of time between primary diagnosis and a clinical event is captured.
- **EUCANCan:** The EUCANCan dictionary builds on the ICGC ARGO dictionary and aligns with both the ICGC ARGO dictionary and mCODE^54^. Mapping of version 1.28 of the ICGC ARGO clinical dictionary against the EUCANCan data model reveals 70% of the core clinical fields in ARGO can be mapped to a field in EUCANCan.
- **NCI’s Genomic Data Commons (GDC):** The NCI’s Genomic Data Commons (GDC) data model is a graph-based model used to organize and manage cancer data ingested by the GDC including clinical data^55^. The GDC data model and ARGO data model share good interoperability, where almost 65% of the core clinical fields in ARGO can be mapped to a field in GDC, with 38% of these ARGO core fields having a partial mapping to a GDC field. Additionally, 26 extended ARGO clinical fields can be mapped to a GDC field. ARGO fields that cannot be mapped to GDC are mainly due to differences in granularity such as, the inclusion of additional response to treatment criteria in the ARGO data model, and the ability to use different drug databases to define drug names. (see Table 4 deposited in Figshare^56^).

### Comprehensiveness

A comprehensive evaluation of a cancer patient encompasses both clinical and genomic information which is key to delivering precision medicine to patients. Existing CDMs/projects such as OSIRIS^57^, 1+MG-MDC^39^, mCODE^37^ and GENIE^58^ collect and link genomic alterations with clinical information, but the genomic data is limited by the lack of data harmonization, such as the use of different sequencing panels which can impact the frequency of variants identified^59^. By contrast, the ICGC ARGO data model does not incorporate fields for genomic information since the raw sequencing data is required to be submitted separately alongside the Sample Registration schema and is processed uniformly by the ICGC ARGO computing facility. The Sample Registration schema focuses on quality control and validation by linking the genomic data to the clinical information. This is achieved through the design of the data model, which links data across various domains - specifically clinical, genomic and analyzed datasets. This cross-domain integration is a key strength of the data model, facilitating cross comparison across datasets. Additionally the data model is also donor-centric, offering flexibility in data submission by enabling submission of genomic and clinical data in parallel.

While the data model is not explicitly designed for large language models (LLMs), the data model exhibits several strengths that make it supportive of artificial intelligence (AI) and LLM. These include the use of self-explanatory field names in both machine-friendly and human-readable formats, which helps improve comprehension for LLM. The data model also standardizes units and data formats which reduces ambiguity. As mentioned above, plans to incorporate links to fields/values to ontology concepts such as UMLS, SNOMED and NCIt and ensuring semantic relationships between fields will enable the data to be indexed. The cross-file and conditional validation checks ensure the dataset is comprehensive and internally consistent which is essential for training reliable AI models. The requirement of key core fields helps reduce misinterpretation by LLMs by promoting complete and consistent data records. The data model also enhances the FAIRness (Findability, Accessibility, Interoperability and Reusability) of the data, thereby increasing its suitability for AI/LLM purposes.

While the ICGC ARGO clinical data model is not intended to replace existing cancer data models, its explicit design is uniquely suited for federated multi-institutional precision oncology research on a global scale. Unlike other common data models (Table 1):

- OMOP is flexible and ideal for epidemiological studies but requires disease-specific extensions and lacks deep support for complex cancer treatments.
- mCODE is optimized for EHR interoperability and standardized clinical data exchange, but is limited in capturing longitudinal outcomes and deep genomic data.
- GA4GH Phenopackets provides flexible, ontology-driven modeling, but is less cancer-specific and lacks standardized tracking of treatment response and disease progression.
- GDC excels in genomic data integration but does not provide rich clinical tracking over time.

**Table 1:** Comparative framework of different cancer clinical data models.

Criteria	ICGC ARGO	mCODE	GDC	GA4GH Phenopackets	OMOP
Clinical Domains Collected	Donor, Primary Diagnosis, Specimen, Treatment (chemotherapy, radiation, surgery, immunotherapy, hormone therapy), follow up outcomes, family history, biomarkers, exposure, comorbidity, and basic details about a sample.	Patient, Disease, Outcome, Genomics, Treatment, Assessment.	Demographics, Diagnosis, Pathology, Treatment, Follow Up, Family History, Exposure, Molecular tests.	Diagnosis, Phenotypic features, treatment, progression, clinical history and outcomes.	Enables collection of cancer data using the Oncology Extension.
Genomic data integration	Data model ensures strong integration of genomic data by focusing on clinical data and its relationship to biospecimens through standardized identifier fields.	Captures basic genomic information such as biomarkers and genetic test results.	Genomic data is integrated with clinical data in data model.	Has support for variant interpretation. Primary focus is on linking genetics and phenotypes.	Requires external linkages to enable genomic data integration, but these require more on-going development for use in precision oncology.
Longitudinal clinical data	Collects fields for time intervals of each clinical event. Enables linkage of clinical events with each other using foreign keys in each schema.	Supports longitudinal tracking via FHIR events, but practical implementation depends on EHR.	Collects fields for time intervals but these are not required.	Collects timestamps but does not enforce validation to check for sequence of events.	Data model has the ability to organize events temporally but Oncology Extension is not designed to capture complex cancer treatments or disease trajectories.
Data validation	Data model has rigorous schemas and comes packaged with cross-field validation scripts.	Data model does not include validation scripts. Rather it leverages FHIR standards to enforce basic validation.	Primary validation rules (ie. data type and accepted value checking) are defined in JSON schema, while secondary validations are implemented as custom modules referenced in the dictionary.	Supports schema-level validation but lacks checks for clinical correctness.	Comprehensive data validation done externally from the data model.
Treatment response and disease progression	Disease progression and recurrence are modeled explicitly in the data model with associated time intervals and progression/recurrence types. It also captures the qualitative response to treatment and assessment method, enabling reproducible outcome interpretation.	Captures disease progression as an outcome, but does not always include detailed timing of recurrence or linkage to prior treatments. Captures response status rather than detailed metrics such as RECIST.	Collects tumour response but does not enforce linkage to assessment criteria or include follow up intervals.	Allows encoding of treatment response but does not enforce standard assessment methods or timing of disease progression.	Data model captures disease episodes but explicit response to treatment is not standardized.
Standardization	Relies heavily on controlled terminology (ie. AJCC, ICD-O, Snomed etc).	Leverages FHIR profiles with terminology bindings, promoting clinical data exchange but less structured enforcement.	Data model includes references to external standards.	Uses ontology-based coding such as HPO for phenotypic features but not as tumour-specific.	Relies heavily on controlled terminology.

The ICGC ARGO clinical data model offers comprehensive, high granularity clinical data, particularly suited for representing cancer phenotypes, treatments, and outcomes over time.

### Dynamic/Scalability

The ICGC ARGO Dictionary will continue to evolve and adapt new clinical concepts such as new treatments or new genomic data types over the lifespan of the project. The ICGC ARGO Dictionary is updated periodically to accommodate new clinical concepts or use cases on the basis of feedback from ARGO programs and discussions with the Clinical and Metadata Working Group and uses a versioning system to keep track of changes between different versions of the dictionary. In addition to being able to compare different versions in the Dictionary viewer, we also maintain Dictionary Release Notes at https://docs.icgc-argo.org/docs/release-notes/dictionary-releases which provide details on updates done in each dictionary release. Communication about upcoming or current changes to the data model are done via email to all ARGO programs and also discussed during regular monthly meetings with the Data Coordination and Management Working Group. The overall design of the dictionary enables it to be expanded to incorporate additional schemas and/or new data elements, which is evident with other initiatives such as EuCanImage and MOHCCN which have adopted the ICGC ARGO Dictionary into their CDM and expanded it to accommodate their own projects. In the past, the DCC migrated legacy data for ARGO programs where possible, and requested any additional required clinical data from ARGO programs to fulfil validation. However most of the major updates to the data model involving legacy data migration were done early on, and there have been no breaking changes implemented since early 2023 as the data model has become stable and current updates are to accommodate clinical submissions. Future work includes accommodating imaging data and targeted therapy information.

### Limitations/future work

The ICGC ARGO dictionary was specifically designed to suit the needs and requirements of the ICGC ARGO project, and as with any CDM, there are limitations, but some of these limitations are deliberate to ensure consistency and interoperability. One limitation is noted by Frid *et al*.^28^ that highlighted that the Biomarker table is closed, and does not allow data submitters to add any biomarker concept^28^. While this is true in the sense that data submitters cannot simply add biomarkers as is possible with OMOP using codeable concepts, data submitters can submit a request to the DCC or Clinical & Metadata Working Group regarding new biomarker fields to be added to the dictionary. Some of the biomarker test names used in the dictionary are standardized and mapped to, for example LOINC. However, LOINC does not always provide a list of standardized accepted test values that can be validated against, and this could potentially result in diverse reporting measures that can cause data consolidation issues later. For this reason, we explicitly list biomarkers as fields with accepted values to ensure the biomarker results can be validated to ensure consistency. An example of this is EGFR expression and its interpretation which can be diverse based on a pathologist’s visual scoring or diverse staining. This can result in different scoring methods being used (i.e. 1+, 2+. 3+ or “positive”) which can limit data consolidation^60^. Although our approach to listing individual fields for each biomarker could lead to a large Biomarker table in the dictionary, it does not impact data submitters as they need to submit only the biomarker fields that are applicable to them. More importantly, it promotes consistency among the clinical data records that groups report using the data model.

Since cancer research is an evolving field, it is inevitable that the dictionary will require periodic updates as new treatments, new biomarkers or new cancer subtypes emerge. Some updates can take time to implement in the dictionary and possibly delay the submission of clinical data. This is because the update can impact other aspects of the dictionary or may have implications for clinical data already submitted that needs to be assessed and tested before release. For example, the addition of a new field or term can sometimes require cross-validation against another field, which involves implementing code. An example during earlier dictionary development was the addition of a new tumour grading system, which requires cross-validating the new tumour grades against the value of the “tumour_grading_system” field to ensure that they belong to the specified tumour grading system. Any update to the dictionary undergoes rigorous testing before the dictionary update is deployed, which ensures that clinical data submissions are correctly validated to ensure high quality clinical data collection.

As an example of ever-evolving treatments that need to be considered, we plan to update the dictionary to capture targeted therapy which has seen the continuous development of more precise and personalized targeted treatments such as next generation inhibitors and gene and RNA-based therapies. In the near future we plan to add a new schema for targeted therapy to enable reporting of targeted therapy drugs such as tramedtinib, a classical MEK kinase inhibitor.

Another challenge is ensuring compliance with regulations such as the GDPR, while balancing data sharing and privacy. Certain fields may need to be censored or masked before data sharing, which affects data queries and metrics. Although these regulations pose challenges to data sharing, potential solutions and further guidance are being actively discussed.

## Conclusion

The ICGC ARGO Dictionary has several strengths that make it a valuable resource in cancer research. The Dictionary captures a wide array of clinical data including primary diagnosis, pathological, treatment and family history information, enabling it to support complex analyses across different cancer types. What makes it stand out compared to other CDMs is its collection of high quality clinical data, thanks to stringent validation checks and quality control. By adhering to standardized terminology, the dictionary ensures consistency across ICGC ARGO programs and facilitates interoperability with several other standards including mCODE, GA4GHPenopackets, GDC, 1 + MG-MDC, EuCanImage, EUCanCan and MOHCCN. As a community driven development, the dictionary will continue to evolve through collaboration with researchers around the world, and regular feedback and consensus with experts ensures that it remains up to date with the latest advancements in cancer research to support ICGC ARGO’s goals. By combining these strengths, the ICGC ARGO Dictionary can be leveraged as a powerful resource in precision medicine and is helping global collaboration in cancer research.

## Supplementary information

Table S1

## Data Availability

The ICGC ARGO clinical data model is available in our ICGC ARGO GitHub organization repository (https://github.com/icgc-argo/argo-dictionary) and our Dictionary Viewer at https://docs.icgc-argo.org/dictionary. All data supporting the results of this article have been deposited in Figshare or included in this article as supplementary information. Descriptions of standardized ontologies and terminologies used in the ICGC ARGO data model are summarized in Table S1 as a supplementary table in this article. A table detailing references to standardized terminologies used in the ICGC ARGO Dictionary is available as Table 1 at 10.6084/m9.figshare.30193936. Comprehensive mapping of ICGC ARGO data model against 6 oncology-focused CDMs is available as Table 2 at 10.6084/m9.figshare.30204112. The full field-level mapping between ICGC ARGO data model (v1.28) and mCODE (STU 4.0) is available as Table 3 at 10.6084/m9.figshare.30203965. The full field-level mapping between ICGC ARGO data model (v1.28) and GDC data model (v3.3.0) is available as Table 4 at 10.6084/m9.figshare.30215407. All data are publicly accessible and are shared under a Creative Commons license, CC BY 4.0.

## Code availability

The ICGC ARGO Dictionary along with the code for the Dictionary Viewer is maintained and available under the AGPL-3.0 license from the ICGC ARGO GitHub organization repository at https://github.com/icgc-argo/argo-dictionary, and is available to view at https://docs.icgc-argo.org/dictionary. The documentation is available at https://docs.icgc-argo.org/.
