# Supplementary material for: The ICGC ARGO data dictionary for standardizing global cancer clinical data
Source: Sci Data. 2025 Nov 20;12:1852. doi: 10.1038/s41597-025-06068-4 (PMC12635221; doi:10.1038/s41597-025-06068-4)
Supplement: Supplementary file 1 — Table S1 [file 41597_2025_6068_MOESM1_ESM.doc]

**Supplementary table S1: Description of standardized ontologies and terminologies used in the ICGC ARGO data model**

| **Name** | **Description** | **Dictionary standardization** |
| --- | --- | --- |
| International Classification of Diseases, 10th Revision (ICD-10) | A medical classification list by the World Health Organization containing codes for diseases including cancer.^26^ | Used to specify the cancer_type_code field. |
| International Classification of Diseases for Oncology, 3rd edition (ICD-O-3) | A multi-axial classification of the site, morphology, behaviour, and grading of neoplasms.^27^ | Used to standardize the controlled terminology for primary_site and accepted values for the tumour_histological_type, specimen_anatomic and surgery_site fields |
| RxNorm | Provides normalized names for clinical drugs and links its names to many of the drug vocabularies commonly used in pharmacy management and drug delivery systems^22^ | Used to standardize accepted values for the drug_rxnormcui and drug_name fields. |
| PubChem | A database of chemical molecules.^23^ | Used to standardize accepted values for the drug_id and drug_term fields. |
| KEGG DRUG Database  (KEGG) | A comprehensive drug information resource for approved drugs in Japan, USA and Europe, unified based on the chemical structure and/or the chemical component of active ingredients.^24^ | Used to standardize accepted values for the drug_id and drug_term fields. |
| NCI Thesaurus | A reference terminology used by the National Cancer Institute to specify concepts used in the cancer research community.^61^ | Used to standardize accepted values for the drug_id and drug_term fields. Also commonly used to standardize several controlled vocabulary terms and field definitions. |
| Common Terminology Criteria for Adverse Events (CTCAE) | A set of criteria for the standardized classification of adverse events of drugs and treatment used in cancer therapy.^14^ | Used to standardize controlled terminology in the hematological_toxicity and non-hematological_toxicity and adverse_events fields. |
| RECIST | Response Evaluation Criteria in Solid Tumours^62^ | Used to standardize response_to_treatment values if RECIST was used. |
| Logical Observation Identifiers Names and Code (LOINC) | A universal standard for health measurements, observations and documents.^63^ | Used to standardize several biomarker tests in the Biomarker table. |
| North American Association of Central Cancer Registries (NAACCR) | A professional organization that develops and promotes uniform data standards for cancer registries in North America.^64^ | Used to standardize controlled terminology for lymphovascular_invasion and perineural_invasion fields in the Surgery table, as well as several biomarker tests in the Biomarker table. |
| Cancer Standards Registry and Repository (caDSR) | A database designed by NCI to create and use data standards for cancer research.^65^ | Used to standardize several fields in the dictionary, including field description and controlled terminologies. |
| Unified Medical Language System (UMLS) | Integrates and distributes key terminology, classification and coding standards, and associated resources to promote creation of more effective and interoperable biomedical information systems and services, including electronic health records.^15^ | Used to standardize controlled terminology for surgery_type and several other controlled terminologies in the dictionary. |
| International Cancer Committee on Cancer Reporting (ICCR) | Internationally standardised and evidence based datasets for the pathology reporting of cancer.^13^ | Used to standardize margin related fields in the Surgery table. |
| Cancer Care Ontario Reporting Standard | Standards set by Cancer Care Ontario for regional cancer centres and healthcare organizations.^66^ | Used to standardize controlled terminology for anatomical_site_irradiated in Radiation Therapy table. |
| Orphanet Rare Disease Ontology | The Orphanet Rare Disease Ontology (ORDO) is a structured vocabulary for rare diseases derived from the Orphanet database.^67^ | Used to standardize controlled terminology for genetic_disorders field in Donor table. |
| American Joint Committee on Cancer Staging Classifications (AJCC) | An international system used for staging cancer.^25^ | Used to standardize controlled terminology for fields related to T, N and M categories, as well as stage_group and some fields in the Surgery table (eg. residual_tumour_classification) |
| Systematized Nomenclature of Medicine (SNOMED) | A structured clinical vocabulary designed to be used within an electronic health record (EHR) or electronic medical record (EMR).^68^ | Used to standardize controlled terminology for treatment_intent, outcome_of_treatment and some terms in surgery_type |
| International Association of Cancer Registries (IACR) Standard for Basis of Diagnosis | The International  Association of Cancer Registries recommend the following codes for recording the  “most valid basis of diagnosis”.^69^ | Used to standardize controlled terminology for basis_of_diagnosis field |
| Eastern Cooperative Oncology Group (ECOG) Performance Status Scale | Standard criteria developed by the Eastern Cooperative Oncology Group for measuring how a patient’s disease impacts their daily living abilities.^70^ | Used to standardize controlled terminology for performance_status field. |
| Human Phenotype Ontology (HPO) | Provides a standardized vocabulary of phenotypic abnormalities encountered in human disease.^71^ | Used to standardize controlled terminology for the presenting_symptoms field. |
| Units of measurement ontology | The Units Ontology (UO) provides a standardised description of units of measurement in science.^72^ | Used to standardize units for the drug_dose_units field in the chemotherapy and hormone therapy tables. |

61 Fragoso, G., De Coronado, S., Haber, M., Hartel, F. & Wright, L. Overview and utilization of the NCI Thesaurus. *Comp Funct Genom* **5**, 648–654 (2004). <https://doi.org/10.1002/cfg.445>

62 Therasse, P. et al. New Guidelines to Evaluate the Response to Treatment in Solid Tumors. JNCI: *Journal of the National Cancer Institute* **92**, 205–216 (2000). <https://doi.org/10.1093/jnci/92.3.205>

63 Bhargava, A., Kim, T., Quine, D. B. & Hauser, R. G. A 20-Year Evaluation of LOINC in the United States’ Largest Integrated Health System. *Archives of Pathology & Laboratory Medicine* **144**, 478–484 (2020). <https://doi.org/10.5858/arpa.2019-0055-oa>

64 Thornton ML, (ed). Standards for Cancer Registries Volume II: Data Standards and Data Dictionary, Version 23, 24th ed. Springfield, Ill.: North American Association of Central Cancer Registries, August 2022. <https://apps.naaccr.org/data-dictionary/data-dictionary/version=23/chapter-view/>

65 caDSR II <https://cadsr.cancer.gov/onedata/Home.jsp> [online] (Accessed March 16, 2025)

66 Cancer Care Ontario - Data Book – Information & Reporting Standards. <https://www.cancercareontario.ca/en/data-book-reporting-standards> [online] (Accessed March 16, 2025)

67 Orphanet Rare Disease Ontology. <https://www.ebi.ac.uk/ols4/ontologies/ordo> (Accessed March 16, 2025)

68 Chang, E. & Mostafa, J. The use of SNOMED CT, 2013-2020: a literature review. Journal of the American Medical Informatics Association 28, 2017–2026 (2021). <https://doi.org/10.1093/jamia/ocab084>

69 International Association of Cancer Registries (IACR) Standard for Basis of Diagnosis. <http://www.iacr.com.fr/images/doc/basis.pdf> [online] (Accessed March 16, 2025)

70 Azam, F. et al. Performance Status Assessment by Using ECOG (Eastern Cooperative Oncology Group) Score for Cancer Patients by Oncology Healthcare Professionals. *Case Rep Oncol* **12**, 728–736 (2019). <https://doi.org/10.1159/000503095>

71 Köhler, S. et al. The Human Phenotype Ontology in 2021. *Nucleic Acids Research* **49**, D1207–D1217 (2021). <https://doi.org/10.1093/nar/gkaa1043>

72 Gkoutos, G., Schofield, P. & Hoehndorf, R. The Units Ontology: a tool for integrating units of measurement in science. Database: *The Journal of Biological Databases and Curation* 2012 (2012). <https://doi.org/10.1093/database/bas033>
